# A Simplified Digital Approach to the Treatment of a Postpuberty Patient with a Class III Malocclusion and Bilateral Crossbite

**DOI:** 10.1155/2021/3883187

**Published:** 2021-09-30

**Authors:** Domenico Aiello, Riccardo Nucera, Stefania Costa, Michele Mario Figliuzzi, Sergio Paduano

**Affiliations:** ^1^Department of Health, University Magna Graecia of Catanzaro, Viale Europa, Loc Germaneto, 88100 Catanzaro, Italy; ^2^Department of Biomedical and Dental Sciences and Morphofunctional Imaging, Section of Orthodontics, University of Messina, Italy

## Abstract

Monolateral and bilateral crossbites are amongst the most frequent forms of malocclusion in the world population. The lack of early correction of this type of malocclusion leads to the partial or total ossification of the sutures which then require surgical treatment in adult patients. In recent years, devices on minipalatal screws have noticeably increased the time window in which it is possible to correct these types of alterations. In this case report, we show how it is possible to correct a third-class skeletal malocclusion associated with a posterior bilateral crossbite in a young woman using a rapid expander on miniscrews and fixed orthodontic device to finalise the process. The procedure for the insertion of the palatal screws was aided by the use of a digitally printed surgical guide, and the appliance was applied in the same sitting thanks to the use of a digital flow software and a systematic easy driver. The CBCT scans show how the orthopaedic expansion of the upper maxilla was obtained without any important alterations that damaged the permanent teeth. This case report wishes to demonstrate how easy and predictable it can be to resolve cases of this type with optimal aesthetic and functional results even when body growth has ended.

## 1. Introduction

According to an earlier study of ours, about 13% of young people suffer from bilateral posterior crossbites [1]. Correction of this problem, in the cases where there is a maxillary defect, is by following a therapy with a rapid palatal expander [2–5]. However, this type of appliance is much more effective when used before the peak of growth occurs, but they lead to minor success rates and increased negative consequences to the permanent teeth at postpuberty [6]. In recent years, research has been concentrated on the use of appliances that are partially or completely anchored to the bone (MARPE). These devices seem to reduce to a minimum any dental alterations arising from this form of therapy [7], and from a study of the finished elements carried out by Seong et al., it seems that the distribution of the forces is decisively better in the hybrid types compared with the types that are purely skeletal or purely dental [8]. This type of appliance seems to considerably reduce the therapeutic indications for the surgically assisted expansion (SARPE) reducing the operational risks of this kind of therapy [9]. Based on the information available about implant surgery [10, 11] and the new concepts of digital odontology [12], many firms have begun to develop surgical guidance systems to insert the TADS with a greater control over the positioning of the same [13, 14], thus guaranteeing bicorticality [15, 16] and allowing greater safety of the anatomic structures present. However, these methods still require the taking of an impression that can lead to imprecisions in the manufacture of the devices and do not permit an immediate fitting of the TADs as is usually advised in orthodontics [17, 18]. Methods that permit the insertion of miniscrews and appliances in one sitting with digital planning have been created only very recently, and these reduce the therapeutic risks and speed up the start of the therapy [19].

## 2. Case Report

This case report discusses the treatment of a girl who had reached the end of her growth and was a third-class skeletal case with bilateral crossbite. She was treated with the help of a hybrid expander positioned in one sitting and finished with a fixed therapy extra torque type multibracket following the prescription by Prof. R. H. Roth.

### 2.1. Diagnosis and Aetiology

**Figure 1:**
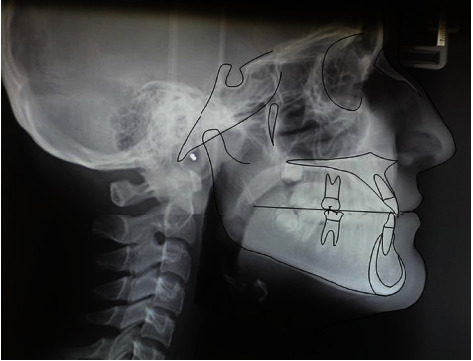
Initial lateral cephalogram.

**Figure 2:**
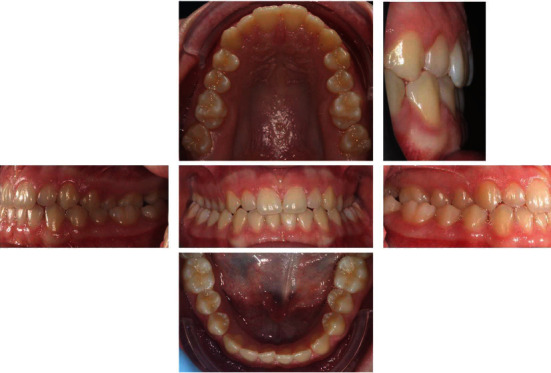
Initial intraoral photographs.

**Figure 3:**
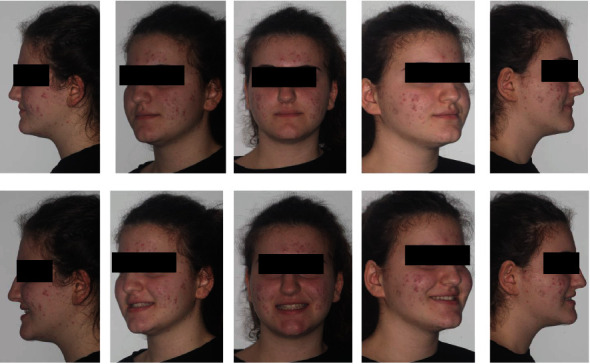
Initial extraoral photographs.

**Figure 4:**
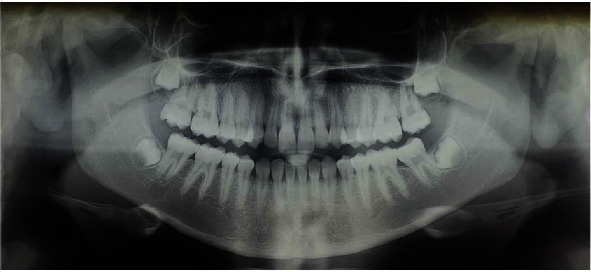
Initial OPT.

**Figure 5:**
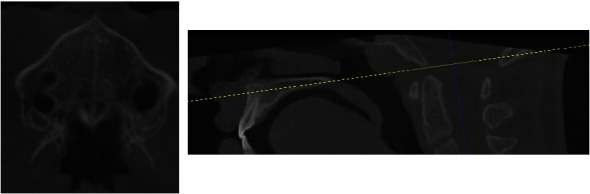
CBCT suture evaluation.

The patient, female, aged 14 years and 6 months whose anamnesis showed that menarche had occurred 2 years and 3 months earlier, did not report any signs or symptoms of gnathological problems [20] and on the OSAS questionnaire was not considered a patient at risk [21]. She presented a dentally compensated Class III skeletal malocclusion and a bilateral crossbite with a complete inversion of the cusp-pit rapport. The cephalometric analysis (Figure 1 and Table 1, carried out using Delta-Dent software, outside format, Spino d'Adda, Italy) brought to light the third-class skeletal malocclusion (AN/Pog-5°) brachyfacial (SN/Go-Gn: 28.7°; Ans-Pns/Go-Gn: 17.5°) characterised by a development deficit of the upper maxilla whilst the mandible was positioned normally and presented the correct dimensions. At a dental level (Figure 2), the patient presented a first-class molar rapport and a canine with upper incisor positioned normally, an important retro inclination of the lower incisors (-1/Go-Gn: 80°) and an increase in the interincisor angle (144.4°). Aesthetically (Figure 3), the patient had a good profile with a minor increase of the nasolabial angle although a slight protrusion of the chin was noticeable given the anatomy of the mandibular symphysis. The upper interincisor line was centred with the median of the face whilst the lower one was deviated by about 2 mm towards the right. The initial OPT did not show any relevant problems (Figure 4). The results of the CBCT scan (Figure 5) showed a partial ossification of the mediopalatine suture rated as category B according to the article by Angelieri et al. [22] but with 2 well-defined lines of ossification for about half of the posterior suture line. All in all, from a dental and periodontal point of view, the patient did not show signs of dental disease or periodontal problems.

### 2.2. Treatment Objectives

In treating this patient, the following objectives were fixed: correction of the bilateral posterior crossbite using orthopaedic expansion procedures avoiding dental compensations; achievement of a first-class canine and molar occlusion with good compensation of the skeletal problems; correction of the overbite and overjet; improvement, or at least nondeterioration of the facial aesthetics; and visibility of the upper incisors when smiling.

### 2.3. The Sequence of the Treatment

**Figure 6:**
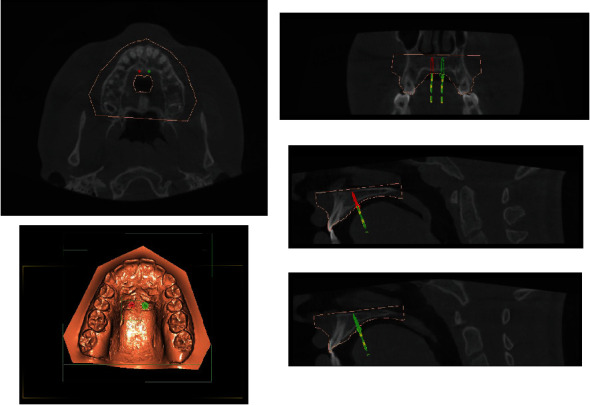
Implants digital planning.

**Figure 7:**
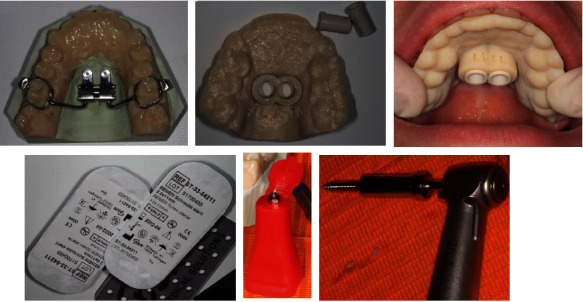
Operative sequence.

**Figure 8:**
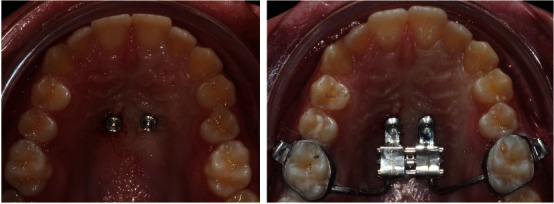
Implants and hybrid RME placed.

**Figure 9:**
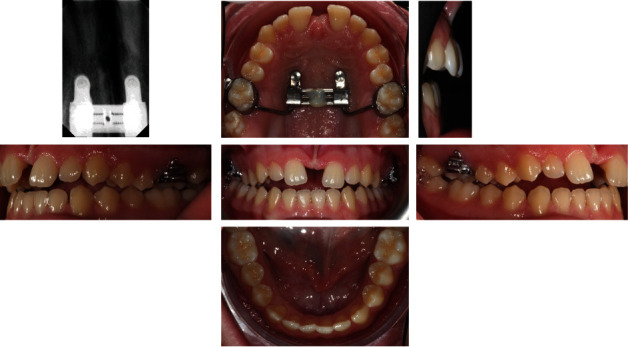
Before expansion treatment.

Given the type of mesiopalatine suture found in the patient [22], in order to ensure that the suture expansion was as controlled as possible to reduce dental effects, we opted for using a hybrid rapid palatal expander on 2 miniscrews, thus allowing the most uniform discharge of the expanding forces following the Easy Driver® protocol; this digitalised procedure gives a clinician the possibility of using a surgical guide for the placement of the screws and a rapid palatal expander which can be positioned and activated right from the first sitting, thus avoiding the need for intermediate dental impressions and consequent imperfections due to the same as well as increase of therapy time. The protocol makes use of a superimposition of Standard Triangulation Language (STL), obtained in this case from the scan of the initial impression and superimposed on the Digital Imaging and Communication in Medicine (DICOM) obtained from the CBTC using the specific Easy Driver® software (Figure 6). With this software (Easy Driver®, Uniontech, via R. Bormioli, 5/A, 43122 Parma, Italy), it is possible to insert the 2 miniscrews following the principal of bicorticality [23] and, using specific 3D stereolithographic dental printers, to print the surgical guide for the insertion of the screws supplied with specifically placed holes for the bushings (as supplied in the implant kit) together with a model to which analogous implants are applied which leads to the preparation of the definitive device together with its fixing screws. During the first operational sitting, the medial and distal separating elastics on the first upper molars were removed, the device was tried out in the oral cavity, and the two screws were positioned (length 11 mm × 2 mm Ø Benefit, PSM, Gunningen, Germany) using an implant micromotor (Surgic XT plus, Nakanishi Inc., 700 Shimohinata, Kanuma-shi, Tochigi, 322-8666, Japan) set at 20 rpm and 25 N/cm torque using the free-drill method without any need for irrigation (Figures 7 and 8). In a separate sitting, the appliance was cemented to the first upper molars using GIC—glass ionomer cement (Ketak Cem, 3M, Maplewood, Minnesota, USA), and following this, it was anchored, using specific tightening screws, onto the TADs. The expansion procedure included 4 turns of the screws immediately after this (0.8 mm) followed by 2 turns every day (=0.4 mm) for a total of 23 days. Fifteen days after the initial activation, a check-up X-ray was taken to assess the opening of the suture (Figure 9). At the end of the expansion procedure, the appliance was blocked with composite resin inside the expansion screws, and the patient was monitored for three months. Once the control period was ended, the bands on the molars and the arms were removed leaving only the expansion screws as the means of restraint. Then, a multibracket appliance was inserted (Ovation, Dentsply Sirona, Charlotte, NC USA) with an extratorque prescription according to the technique of Prof. R.H. Roth to correct the imperfections caused by a slight crowding and the rotations found in the arch, thus favouring a complete and correct engagement between the arches. The extratorque prescription turned out to be a high performer in this case of compensation aimed at increasing the torque of the incisors and easing the dental compensation of the skeletal problem. For posttherapeutic restraint, we used a 33-43 retainer in the lower region and a thermoplastic mask in the upper levels.

## 3. Results of the Treatment

**Figure 10:**
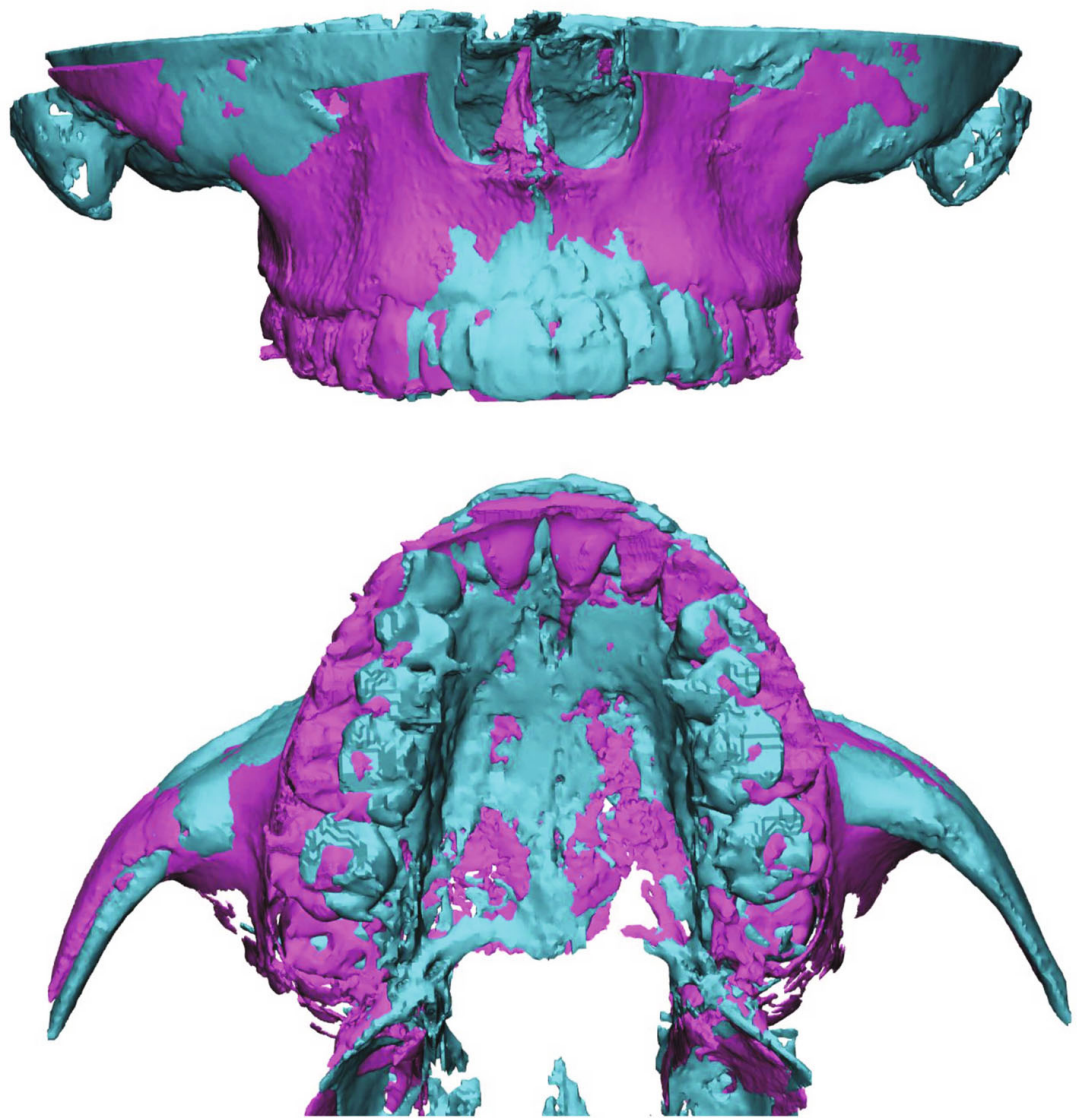
Superimposition of CBTC, light blue pre- and violet postexpansion.

**Figure 11:**
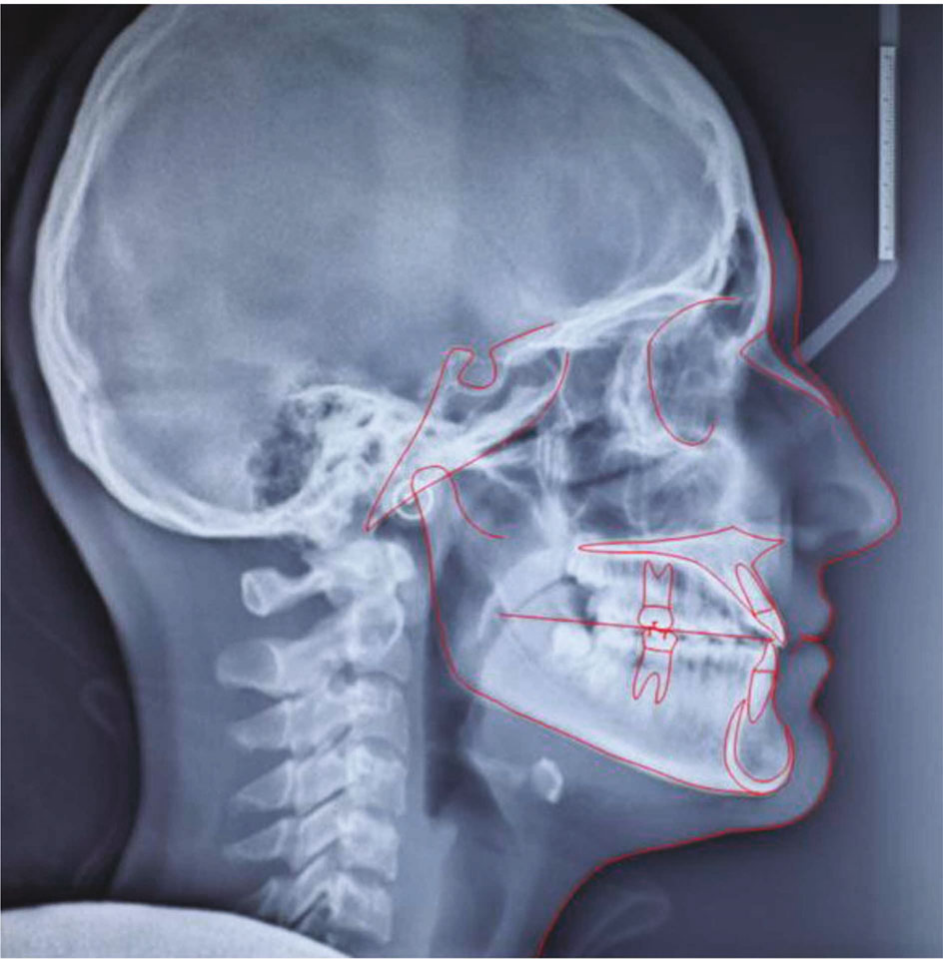
Final lateral cephalogram.

**Figure 12:**
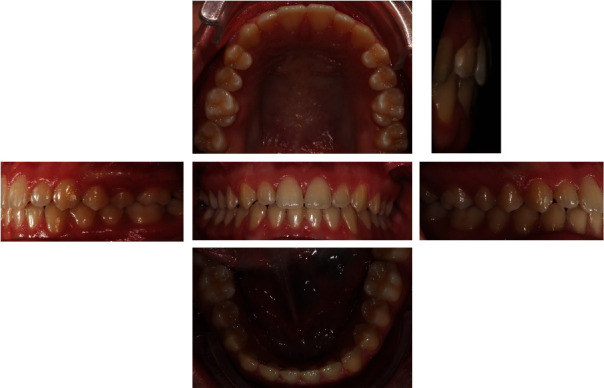
Final intraoral photographs.

**Figure 13:**
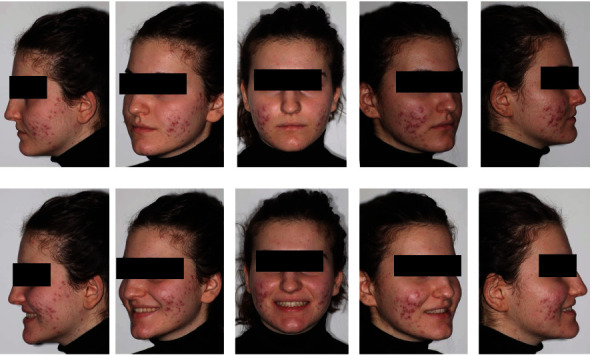
Final extraoral photographs.

**Figure 14:**
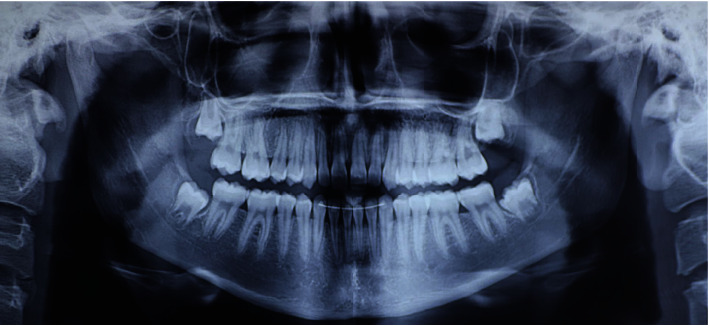
Final OPT.

**Figure 15:**
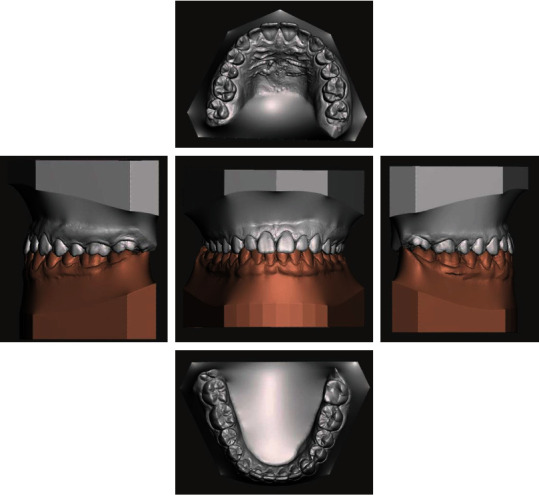
Final virtual models.

The total duration of the therapy was about 18 months. The bilateral crossbite was completely resolved with the skeletal expansion procedure. Analysis on superimposition of the CBCT prior to treatment and after treatment (Figure 10) shows a real skeletal expansion with a minimum effect on the dental elements. The patient finished the therapy with a correct rapport for the molars and the canines and an excellent engagement of the arches. The cephalometric data (Figure 11 and Table 2) report a vertical increase (SN/Ans-Pns from 11,2° to 6,7°; Ans-Pns/Go-Gn from 17,5° to 21,3°) but most of all an excellent dental compensation with an increase in the inclination of the inferior incisors, normalisation of the position of the upper incisors, and a reduction of the interincisal angle (from 147° to 138.9°). However, the patient presented a minor dental compensation such as a minor reduction of the overbite due to an increased Bolton ratio with lateral parts that are reduced and the profile remained unchanged compared with that at the start of the therapy (Figures 12 and 13). The final opt shows good root parallelism (Figure 14). On virtual models, it is possible to evaluate the perfect interdigitation between the arches (Figure 15). To encourage a further aesthetic improvement, the patient was advised to have reductive mentoplasty surgery. This procedure would have promoted a noticeable reduction of the projection of the chin creating greater harmony in the profile. Unfortunately, the patient did not accept this proposal.

## 4. Discussion

**Figure 16:**
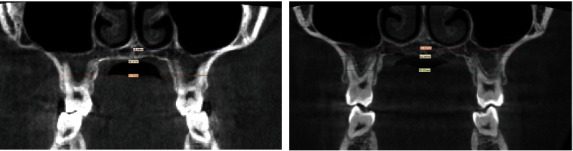
Expansion virtual evaluation.

**Figure 17:**
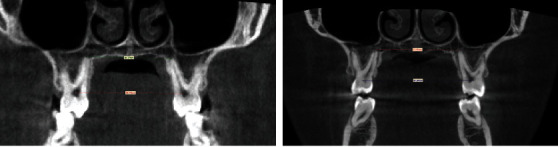
First molar torque evaluation.

**Figure 18:**
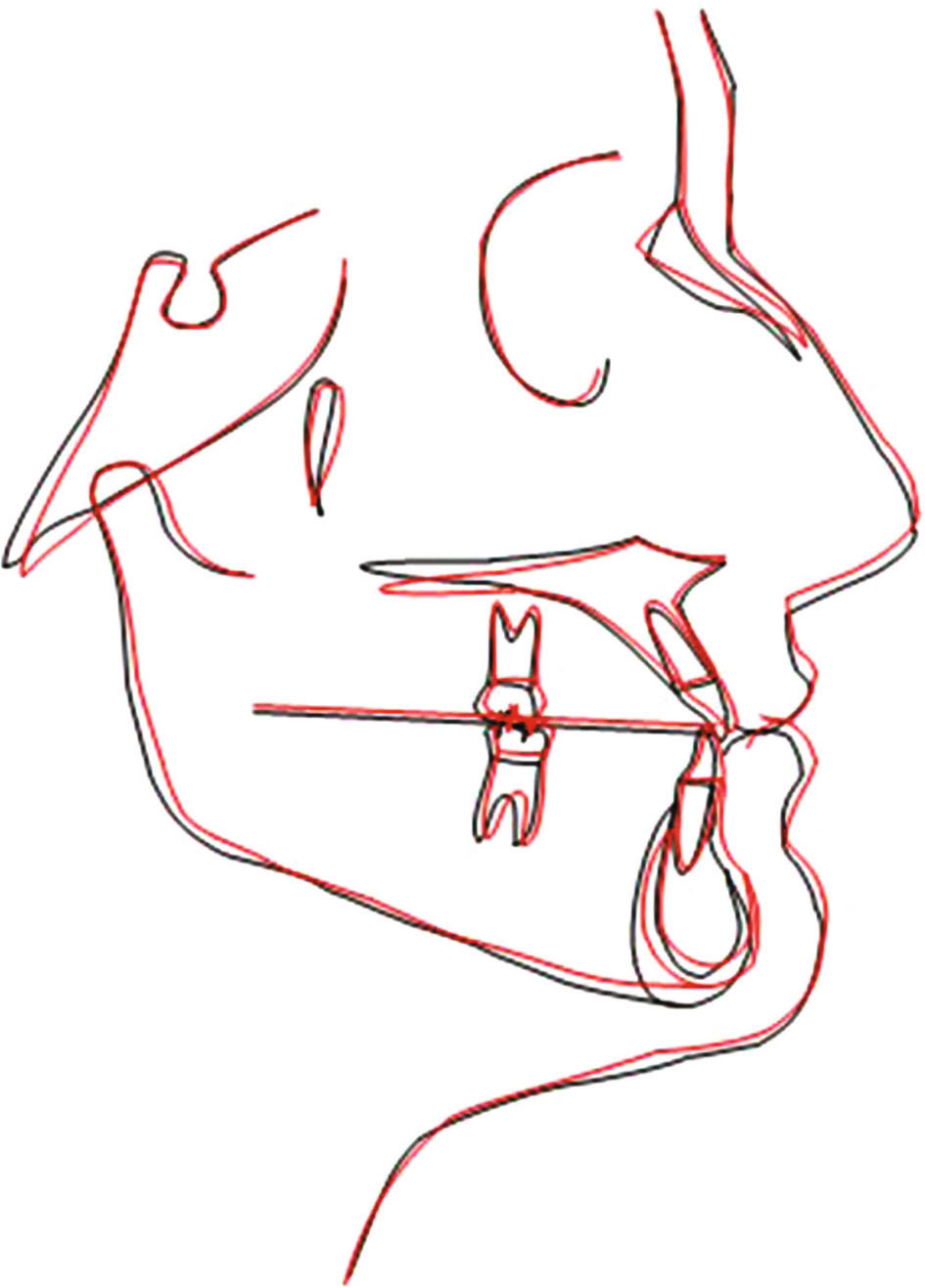
Cephalometric superimposition.

The rapid expansion of the palate is certainly one of the most used and studied practices in orthodontics. The epidemiological prevalence of monoliteral crossbites has been estimated between 8.5 and 11.6% [24, 25] whilst that of bilateral crossbites stands between 1.19 and 5% [25, 26], and specifically, in the Italian population, the prevalence for monolateral crossbites stands at 10.2% and for bilateral ones at 3.1% [1]. If this alteration is not diagnosed and treated early in life, it can provoke skeletal defects that then become structural ones. According to Baccetti et al. [6] the correction of a crossbite achieves the best results when carried out early, before the growth peak occurs. In adults and older adolescents, clinicians very often require the auxiliary of an expansion therapy that is surgically assisted in order to guarantee certain opening of the suture and to avoid excessive collateral effects on the dentition, especially in female patients [27]. In recent years, however, there has been an exponential increase in the use of expanders on miniscrews as an alternative to surgically assisted expansion in adult patients [28] and patients at the end of their growth with reasonably acceptable results even in cases of patients aged 35 years when associated with corticopuncture [29]. Thanks to the Easy Driver® protocol, it was, in this case, possible to insert the TADs and the appliance in one sitting without any risks to the anatomic structures, thus considerably speeding up the start of the therapy, reducing discomforts and promoting a procedure that was not operator-dependent that could be used by less expert people. The complete resolution of the other problems of dental nature were obtained with a finishing phase which, thanks to an extratorque prescription, increases the torque to 17° on the central incisors, 10° on the lateral ones, and 3° on the canines, allowing a more rapid compensation of the anterior sector restoring a correct rapport of the overbite and overjet, the correction of the rotations, the recentring of the lower median line, and the achievement of a good occlusion and aesthetics in only a few months (about 12). The superimposition of the CBTC prior to and immediately after the therapy was carried out with the “global registration” function of the software 3-matic Medical (Mimics Innovation Suite, Materialise Technologielaan 15 3001, Leuven, Belgium), and a clear expansion of the upper mandible can be clearly shown as being the results of this treatment protocol. Although the dental assessment was affected by the subsequent orthodontic therapy, it shows the resolution of the crossbite due to the widening of the diameter of the maxillary bone, a sign of a clear orthopaedic expansion without creating any fenestrations at the root levels of the upper molars. Following the article by Lin et al. [30], the linear assessment of expansion was carried out using the Mimics software (Mimics Innovation Suite, Materialise Technologielaan 15 3001, Leuven, Belgium); this was carried out on the coronal section level with the first upper molars along the NF line (maxillary width tangent to the nasal floor), along the HP line (maxillary width level with the lower tangent of the hard palate), and along the HP5 line (5 mm lower than the HP line). The values obtained (Figure 16) show a marked increase at the level of the HP lines which pass from 58 to 62.5 mm with an increase of about 5 mm and the HP5 line that passes from 54 to 59.7 mm with an increase of about 6 mm, whilst there was a paradoxical reduction in the NF line that changed from 67.5 to 71 mm probably due to a lowering of the floor of the nasal choanae. At the dental level (Figure 17), however, it is evident that an important increase in the distance between the central point of the dental pulp of the first upper molars, the PC line, which changes from 38.7 to 47.8 mm with an increase of nearly 9 mm which, associated with an increase in the linear distance between the apices of the palatine roots of the first upper molars, and the RA line that changes from 30.3 to 37.4 mm with an increase of about 7 mm denotes a movement that is more corporal than an increase in the molar torque. The superimposition of the traces shows that there was a mainly dental effect without great verticality changes (Figure 18).

## 5. Conclusions

The presentation of this case shows how simple and practical the treatment of cases with contracted palates can be even in patients who have passed their peak in growth a long time before starting treatment, going on to manage, in a predictable and practical way, skeletal class III malocclusions with the presence of bilateral crossbites and allowing an orthopaedic resolution of the same without transversal compensations. Today, the use of protocols that are not operator-dependent, such as these, makes therapies of this kind both predictable and trustworthy even for operators with little surgical experience. This makes the operation simple, and it respects the anatomic limits, promoting an adequate expansion without compensatory risks or treatments that are surgically assisted.

## Figures and Tables

**Table 1:** Initial cephalometric values.

*Sagittal skeletal relations*		
Maxillary position S-N-A	78.8°	82° ± 3.5°
Mandibular position S-N-Pg	83.9°	80° ± 3.5°
Sagittal jaw relation A-N-Pg	-5.1°	2° ± 2.5°
*Vertical skeletal relations*		
Maxillary inclination S-N/ANS-PNS	11.2°	8° ± 3.0°
Mandibular inclination S-N/Go-Gn	28.7°	33° ± 2.5°
Vertical jaw relation ANS-PNS/Go-Gn	17.5°	25° ± 6.0°
*Dentobasal relations*		
Maxillary incisor inclination 1-ANS-PNS	118.1°	110° ± 6.0°
Mandibular incisor inclination 1-Go-Gn	80.0°	94° ± 7.0°
Mandibular incisor compensation 1-A-Pg (mm)	-0.6	2 ± 2.0
*Dental relations*		
Overjet (mm)	3.0	3.5 ± 2.5
Overbite (mm)	1.4	2 ± 2.5
Interincisal angle 1/1	144.4°	132° ± 6.0°

**Table 2:** Final cephalometric values.

*Sagittal skeletal relations*		
Maxillary position S-N-A	76.9°	82° ± 3.5°
Mandibular position S-N-Pg	83.9°	80° ± 3.5°
Sagittal jaw relation A-N-Pg	-7.1°	2° ± 2.5°
*Vertical skeletal relations*		
Maxillary inclination S-N/ANS-PNS	6.7°	8° ± 3.0°
Mandibular inclination S-N/Go-Gn	28.1°	33° ± 2.5°
Vertical jaw relation ANS-PNS/Go-Gn	21.3°	25° ± 6.0°
*Dentobasal relations*		
Maxillary incisor inclination 1-ANS-PNS	117.4°	110° ± 6.0°
Mandibular incisor inclination 1-Go-Gn	82.4°	94° ± 7.0°
Mandibular incisor compensation 1-A-Pg (mm)	+0.8	2 ± 2.0
*Dental relations*		
Overjet (mm)	3.4	3.5 ± 2.5
Overbite (mm)	0.4	2 ± 2.5
Interincisal angle 1/1	138.9°	132° ± 6.0°

## References

[1] Paduano S., Rongo R., Bucci R. (2018). Is there an association between various aspects of oral health in southern Italy children? An epidemiological study assessing dental decays, periodontal status, malocclusions and temporomandibular joint function. *European Journal of Paediatric Dentistry*.

[2] Giudice A. L., Spinuzza P., Rustico L., Messina G., Nucera R. (2020). Short-term treatment effects produced by rapid maxillary expansion evaluated with computed tomography: a systematic review with meta-analysis. *The Korean Journal of Orthodontics*.

[3] Zuccati G., Casci S., Doldo T., Clauser C. (2013). Expansion of maxillary arches with crossbite: a systematic review of RCTs in the last 12 years. *European Journal of Orthodontics*.

[4] Huang J., Li C. Y., Jiang J. H. (2018). Facial soft tissue changes after nonsurgical rapid maxillary expansion: a systematic review and meta-analysis. *Head & Face Medicine*.

[5] Lagravere M. O., Major P. W., Flores-Mir C. (2005). Long-term skeletal changes with rapid maxillary expansion: a systematic review. *The Angle Orthodontist*.

[6] Baccetti T., Franchi L., Cameron C. G., McNamara J. A. (2001). Treatment timing for rapid maxillary expansion. *The Angle Orthodontist*.

[7] Copello F. M., Marañón-Vásquez G. A., Brunetto D. P. (2020). Is the buccal alveolar bone less affected by mini-implant assisted rapid palatal expansion than by conventional rapid palatal expansion?- A systematic review and meta-analysis. *Orthodontics & Craniofacial Research*.

[8] Seong E. H., Choi S. H., Kim H. J., Yu H. S., Park Y. C., Lee K. J. (2018). Evaluation of the effects of miniscrew incorporation in palatal expanders for young adults using finite element analysis. *The Korean Journal of Orthodontics*.

[9] Carvalho P. H. A., Moura L. B., Trento G. S. (2020). Surgically assisted rapid maxillary expansion: a systematic review of complications. *International Journal of Oral and Maxillofacial Surgery*.

[10] Bover-Ramos F., Viña-Almunia J., Cervera-Ballester J., Peñarrocha-Diago M., García-Mira B. (2018). Accuracy of implant placement with computer-guided surgery: a systematic review and meta-analysis comparing cadaver, clinical, and in vitro studies. *The International Journal of Oral & Maxillofacial Implants*.

[11] Laleman I., Bernard L., Vercruyssen M., Jacobs R., Bornstein M., Quirynen M. (2017). Guided implant surgery in the edentulous maxilla: a systematic review. *The International Journal of Oral & Maxillofacial Implants*.

[12] Lo Giudice A., Rustico L., Ronsivalle V. (2020). A full diagnostic process for the orthodontic treatment strategy: a documented case report. *Dental Journal*.

[13] Cassetta M., Altieri F., Di Giorgio R., Barbato E. (2018). Palatal orthodontic miniscrew insertion using a CAD-CAM surgical guide: description of a technique. *International Journal of Oral and Maxillofacial Surgery*.

[14] Maino B. G., Paoletto E., Lombardo L 3rd, Siciliani G. (2016). A three-dimensional digital insertion guide for palatal miniscrew placement. *Journal of Clinical Orthodontics*.

[15] Brettin B. T., Grosland N. M., Qian F. (2008). Bicortical vs monocortical orthodontic skeletal anchorage. *American Journal of Orthodontics and Dentofacial Orthopedics*.

[16] Poorsattar-Bejeh Mir A. (2017). Monocortical versus bicortical hard palate anchorage with the same total available cortical thickness: a finite element study. *Journal of Investigative and Clinical Dentistry*.

[17] Manni A., Cozzani M., Tamborrino F., De Rinaldis S., Menini A. (2011). Factors influencing the stability of miniscrews. A retrospective study on 300 miniscrews. *European Journal of Orthodontics*.

[18] Migliorati M., Drago S., Gallo F. (2016). Immediate versus delayed loading: comparison of primary stability loss after miniscrew placement in orthodontic patients-a single-centre blinded randomized clinical trial. *European Journal of Orthodontics*.

[19] de Gabriele O., Dallatana G., Riva R., Vasudavan S., Wilmes B. (2017). The easy driver for placement of palatal mini-implants and a maxillary expander in a single appointment. *Journal of Clinical Orthodontics*.

[20] Iodice G., Danzi G., Cimino R., Paduano S., Michelotti A. (2013). Association between posterior crossbite, masticatory muscle pain, and disc displacement: a systematic review. *European Journal of Orthodontics*.

[21] Paduano S., Paduano F. P., Aiello D. (2019). OSAS in developing age: screening of a southern Italy population. *European Journal of Paediatric Dentistry*.

[22] Angelieri F., Cevidanes L. H. S., Franchi L., Gonçalves J. R., Benavides E., McNamara Jr J. A. (2013). Midpalatal suture maturation: classification method for individual assessment before rapid maxillary expansion. *American Journal of Orthodontics and Dentofacial Orthopedics*.

[23] Lee R. J., Moon W., Hong C. (2017). Effects of monocortical and bicortical mini-implant anchorage on bone-borne palatal expansion using finite element analysis. *American Journal of Orthodontics and Dentofacial Orthopedics*.

[24] Gungor K., Taner L., Kaygisiz E. (2016). Prevalence of posterior crossbite for orthodontic treatment timing. *The Journal of Clinical Pediatric Dentistry*.

[25] da Silva Filho O. G., Santamaria M., Capelozza Filho L. (2007). Epidemiology of posterior crossbite in the primary dentition. *The Journal of Clinical Pediatric Dentistry*.

[26] Souza L. A., Elmadjian T. R., Dias R. B. ., Coto N. P. (2011). Prevalence of malocclusions in the 13-20-year-old categories of football athletes. *Brazilian Oral Research*.

[27] Jimenez-Valdivia L. M., Malpartida-Carrillo V., Rodríguez-Cárdenas Y. A., Dias-Da Silveira H. L., Arriola-Guillén L. E. (2019). Midpalatal suture maturation stage assessment in adolescents and young adults using cone-beam computed tomography. *Progress in Orthodontics*.

[28] Brunetto D. P., Sant’Anna E. F., Machado A. W., Moon W. (2017). Non-surgical treatment of transverse deficiency in adults using microimplant-assisted rapid palatal expansion (MARPE). *Dental Press Journal of Orthodontics*.

[29] Suzuki S. S., Braga L. F. S., Fujii D. N., Moon W., Suzuki H. (2018). Corticopuncture facilitated microimplant-assisted rapid palatal expansion. *Case Reports in Dentistry*.

[30] Lin L., Ahn H. W., Kim S. J., Moon S. C., Kim S. H., Nelson G. (2015). Tooth-borne vs bone-borne rapid maxillary expanders in late adolescence. *The Angle Orthodontist*.

